# EnzyMiner: automatic identification of protein level mutations and their impact on target enzymes from PubMed abstracts

**DOI:** 10.1186/1471-2105-10-S8-S2

**Published:** 2009-08-27

**Authors:** Süveyda Yeniterzi, Uğur Sezerman

**Affiliations:** 1 Faculty of Engineering and Natural Sciences, Sabanci University, Istanbul 34956, Turkey

## Abstract

**Background:**

A better understanding of the mechanisms of an enzyme's functionality and stability, as well as knowledge and impact of mutations is crucial for researchers working with enzymes. Though, several of the enzymes' databases are currently available, scientific literature still remains at large for up-to-date source of learning the effects of a mutation on an enzyme. However, going through vast amounts of scientific documents to extract the information on desired mutation has always been a time consuming process. In this paper, therefore, we describe an unique method, termed as EnzyMiner, which automatically identifies the PubMed abstracts that contain information on the impact of a protein level mutation on the stability and/or the activity of a given enzyme.

**Results:**

We present an automated system which identifies the abstracts that contain an amino-acid-level mutation and then classifies them according to the mutation's effect on the enzyme. In the case of mutation identification, MuGeX, an automated mutation-gene extraction system has an accuracy of 93.1% with a 91.5 F-measure. For impact analysis, document classification is performed to identify the abstracts that contain a change in enzyme's stability or activity resulting from the mutation. The system was trained on lipases and tested on amylases with an accuracy of 85%.

**Conclusion:**

EnzyMiner identifies the abstracts that contain a protein mutation for a given enzyme and checks whether the abstract is related to a disease with the help of information extraction and machine learning techniques. For disease related abstracts, the mutation list and direct links to the abstracts are retrieved from the system and displayed on the Web. For those abstracts that are related to non-diseases, in addition to having the mutation list, the abstracts are also categorized into two groups. These two groups determine whether the mutation has an effect on the enzyme's stability or functionality followed by displaying these on the web.

## Background

Enzymes are mostly protein based biomolecules that accelerate the rate of chemical reactions in a living organism. Enzymes are made of amino acids whose unique characteristic composition enables them to have different functionalities and also making them work efficiently at stable conditions, such as optimum temperature and pH. Thus, any mutation that occurs in this amino acid sequence may change the enzyme's 3D-structure, catalytic activity or stability, or even making the enzyme completely non-functional. Therefore, the knowledge of mutations and their impacts are of crucial importance in order to completely understand mechanisms of enzymes' functionality, and stability.

Many experimental studies have focused on finding the effects of mutations on enzymes. For example, many studies have aimed to create enzymes with novel properties such as designing hyperthermophilic enzymes [1] or expanding the substrate specificity of an enzyme [2]. The published results of these projects provide scientific information for researchers who are engaged in finding an impact of mutation on an enzyme. Though many databases are available on the nomenclature of enzymes [3,4] or structure and function [5-11], to our knowledge only BRENDA (BRaunschweig ENzyme Database) [12,13], the largest manually curated enzyme-specific information system, contains an information on engineered enzymes and their effects on the enzyme's catalytic activity while directly referring to scientific literature. Manually curated databases are both slow and expensive for extracting information from scientific literature. There is a need for an efficient automatic extraction method that allows accessing relevant information rapidly with great efficiency, and possibly at any time.

With the latest developments in information extraction, biomedical term recognition has become an important area for researchers. Dictionary-based, rule-based, and machine learning-based approaches are used to extract names of genes, proteins and other cellular substances [14-16]. Several systems have already been developed for automatic extractions of mutations from biomedical literature. MuteXt, for example, developed by Horn et al. [17] is one of the initial works that focused on extracting single point mutations from scientific literature. Moreover, a gold standard data set [18] is created for comparing the performance of mutation extraction systems and systematic evaluations [18,19] for these systems are developed with a precise definition of evaluation metrics. The next step in mutation informatics is finding relation of mutations to other biological entities such as genes or proteins.

Rebholz-Schuhmann et al. [20] developed MEMA, which extracts disease-related mutation-gene pairs from Medline abstracts. In MEMA, identification of both gene names and mutations are based on regular expressions compiled into two different finite state automations. If the abstract or the sentence that the mutation is extracted from contains only one gene name, the detected mutation is associated to that particular gene. However, if there is more than one gene name, the MEMA uses syntactical rules and proximity parameters as a criterion for decision. MuGeX (Mutation Gene eXtractor) uses a similar approach developed by Erdogmus et al. [21] in order to extract disease related protein mutations. MuGeX makes use of regular expressions in identifying possible mutations. However, it also handles ambiguous mutation citations by using machine learning techniques. For gene name identification, it uses a dictionary-based approach and then associates the extracted entities according to proximity measures.

For mutation-protein associations, Lee et al. [22] developed Mutation GraB, which identifies mutations using regular expressions, similar to the previous methods [20,21]. Protein identification is also performed with regular expressions, which search for a dictionary of protein names and synonyms. Lastly, Mutation GraB uses graphs in which shortest-distance search and word bigram analysis are used in order to find the associations between mutations and proteins. MutationMiner which is developed by Baker et al. [23,24] follows a different approach than the previous systems. It mainly focuses on associations between mutations and protein structure visualizations using NLP techniques. The system identifies the proteins and mutations in the form of name entities and if cited in the same sentence, the MutationMiner associates them to one another. Moreover, this system has been improved of late with the support of biological ontologies which make mutation annotations available in a semantically consistent format, and with the OWL ontology which enables the automated means of accessing knowledge possible [25,26].

The above information extraction techniques became necessary because of the increased number of electronic documents. At the same time, however, the task to classify these documents based on their contents makes document classification an important field for researchers. Especially after integrating machine learning techniques to document classification, its accuracy has now became comparable to the less than 100% accuracy of human expertise [27,28]. Therefore, because of growing interest and high accuracy rates, document classification has been used in different applications such as document organization [27,29], word sense disambiguation [30] or web document classification [31,32].

Although the above works make it possible to associate mutations to other biological entities, the experimental results documented in scientific literature cannot be extracted with the techniques discussed above. Therefore, in case of mutation informatics, the next step is to extract information describing the effects of the mutations [17,19,33]. The writers of this paper developed EnzyMiner, which is capable of automatically extracting protein mutations from PubMed [34] abstracts for a given enzyme and classifying their impact on the enzyme's functionality and stability. In the case of mutation identification, the information extraction and document classification methods are used. For impact analysis, document classification techniques are again used for identifying the abstracts that contain a change in the stability conditions or catalytic activity of an enzyme resulted from a mutation.

## Methods

### Algorithm

#### Mutation extraction

In this work, we focus on amino acid level protein mutations. In order to extract this kind of mutation from PubMed abstracts, we used MuGeX [21]'s Mutation Extraction and Disambiguation Modules which are based on regular expressions and machine learning techniques. A set of 20 patterns was formed using phrases that contain protein mutations for regular expression matching in MuGeX. Next, pattern matching was applied to each sentence of the abstracts in order to identify mutations.

One major drawback of using regular expressions is that they are too general to be able to capture only the protein level mutations. A nucleotide mutation such as G32A, or the name of a strain or a cell line, such as H4S may easily be misinterpreted as a protein mutation. In order to eliminate such ambiguities, MuGeX makes use of machine learning techniques. Document classification on topic and content sections of these abstracts identifies them as to whether or not a protein mutation is present.

#### Seperating disease related abstracts

Enzymes are essential for a number of functions in the cell; therefore, any malfunctioning of an enzyme caused by a mutation may be found in relation to a disease. Thus, many research projects are conducted to find the relation between enzyme mutations and diseases. Since, MEDLINE is the largest component of PubMed, all the abstracts of these medical projects are included in the PubMed database. Therefore, when we download all the abstracts that contain the term "mutation" and a specific enzyme name, the medical abstracts are also accompanied in the downloaded information. However, these abstracts contains the general focus on the mutations' effects on the disease development, but not on the functionality or the stability of enzymes. For instance, lipoprotein lipase hydrolyzes lipids in lipoproteins. Mutations in the gene encoding for lipoprotein lipase can lead to lipoprotein lipase deficiency that in turn leads to an increase in the levels of triglycerides in the bloodstream. Therefore, many abstracts that contain both the words "mutation" and "lipase" are about research aiming to find the specific mutations that cause this disease. Similarly, the disease related abstracts do not contain the information on direct impact of the mutation on the enzyme functionality or stability. For that reason, including these abstracts into our classification will mislead our results. In order to prevent corruption of the data, we initially classify the abstracts into two groups: (a) disease related abstracts and (b) non-disease related abstracts. Dictionary based approaches [35] that make use of Medical Subject Headings (MeSH) or Unified Medical Language System (UMLS) are previously used for identifying disease related abstracts. On the other hand, various document classification algorithms are also employed for clinical text classification between disease related documents such as clinical patient records [36]. In EnzyMiner, we used document classification approach for grouping our abstracts into disease related or non-disease related abstracts. Therefore, user is giving the option of choosing disease related or non-disease related abstracts, and base of their selection a list of mutations and their impacts on the enzyme are displayed on the web with direct references to the relevant scientific literature.

#### Impacts of mutation

Enzymatic reaction rates depend on physico-chemical conditions, such as pH and temperature. When these conditions are optimum, the enzyme attains its maximal activity. However, at high temperatures or extreme pH changes, the enzyme may become inactive as a result of denaturation. Stability is the condition in which an enzyme can maintain its structural conformation and activity, yet, a mutation may cause changes in these conditions. For instance, a mutation that decreases the molecular flexibility of an enzyme sometimes may lead to higher thermostability, or a mutation that replaces Glycine with a basic or acidic residue may cause significant changes in the enzyme's optimum pH. Therefore, to understand the governing rules of protein structure stability, researchers carry out mutation studies and check the impact on stability at different temperatures and pH.

Likewise, an enzyme's functionality is also affected by mutations. Unlike most of the chemical catalysts, enzymes are highly selective to their substrates and this specificity is mainly determined by three dimensional coordinates of the active site. Therefore, a mutation that modifies the conformation of an enzyme, especially the catalytic site, may cease its function. A mutation that changes the spatial coordinates of the active site can change enzyme specificity for a specific molecule. On the other hand, all the mutations may not necessarily have a significant effect on the enzyme's stability conditions or catalytic functions. There are experimental results, for example, which show that some mutations have no impact. The abstracts that include only these kinds of mutations have to be filtered out since our main purpose is to find the abstracts that contain impacts of mutations on enzymes. We represented this challenge as a classification problem and removed the abstracts that are classified as no change abstracts from our data set.

### System architecture

**Figure 1:**
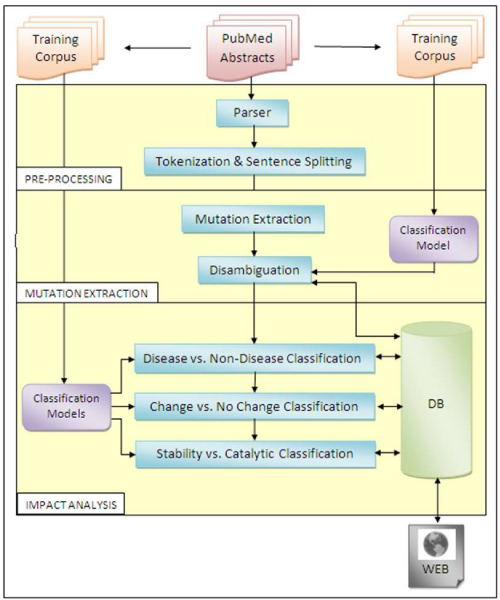
**A schematic illustration of the EnzyMiner system**. DB: database.

The system architecture of EnzyMiner is shown in Figure 1. Our system is composed of three stages: (a) Preprocessing, (b) Mutation Extraction and (c) Impact Analysis. In this work, we focused on the abstracts from PubMed. Abstracts published after January 1, 2000, which contain an enzyme and the possible variations of the term "mutation" are downloaded. In order to include possible variations of "mutation" into our search query, a truncated form of word "mutation" was used. In the case of enzymes, we only used the trivial names in our search queries that supposedly used by all researchers. Further, including EC numbers to our search seems unnecessary since in the abstracts EC numbers are rarely used as the extra information near the enzymes' common names. Since our initial aim was to identify PubMed articles that are focused on a specific enzyme and possible protein mutations, we performed our search restricted to only the 'title' and the 'abstract' sections. If the searched words are not found in the abstract but found in the other fields, such PubMed entry was not downloaded.

While in the Preprocessing stage, the downloaded PubMed abstracts are first parsed, and then tokenization followed by sentence splitting operations were performed on these parsed abstracts for the successive stages.

As for as the mutation extraction stage is concerned, we used MuGeX [21] which is developed by our group to extract disease related mutation-gene pairs. MuGeX consists of two stages and each stage has several modules. Since this work requires us to identify only protein mutations, we therefore used Mutation Extraction and Disambiguation Modules of MuGeX. Mutation Extraction Module makes use of pattern matching with regular expressions to find the possible protein mutations from the text. All of the regular expressions are based on one pattern that starts with an amino acid as the one letter code followed by a number, and ends with another amino acid as another one letter code (e.g. W16A). By making modification to this pattern, a set of 20 patterns was formed in order to find remaining mutation patterns.

Regular expressions are able to identify protein mutations. However, as indicated previously, the main challenge in amino-acid-level mutation extraction is to distinguish actual protein mutations from nucleotide mutations, or mutation like terms such as the name of a strain or a cell line. Disambiguation Module of MuGeX eliminates these kinds of ambiguities by using document classification as a word sense disambiguation application. This module uses Rainbow [37], one of the front ends of the Bow library designed for document classification, so as to decide the abstracts that contain potentially ambiguous mutations. A training benchmark that consists of 3,600 randomly selected Medline abstracts is formed and all of these abstracts were labeled by experts. Out of these, a total of 2771 abstracts contained protein mutations while the remaining abstracts contain either nucleotide mutations or biological entities that are cited with mutation-like terms. Using these labeled abstracts, the Disambiguation Module trains a model for classification while processing the abstracts by performing stemming, alphanumeric tokenization and considering word bigrams.

After the preprocessing stage, EnzyMiner identifies all possible protein mutations in the downloaded abstracts using the Mutation Extraction Module. Then the ambiguous abstracts are queried to the Disambiguation Module where they are classified using Naive Bayes algorithm, which is trained with the above model. At the end of this stage, abstracts that contain protein mutations are queried to the classification modules of the Impact Analysis Stage for further classification.

**Figure 2:**
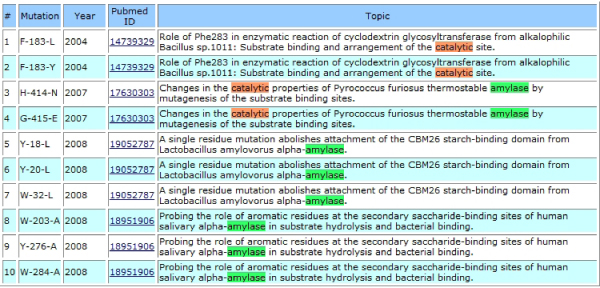
**Abstracts that contain a change in the catalytic activity of amylase resulted from a protein mutation**.

**Figure 3:**
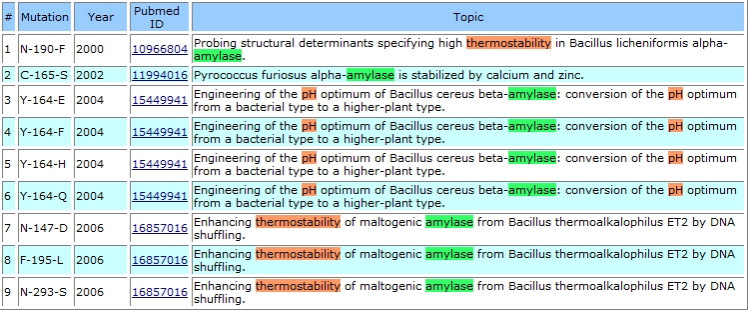
**Abstracts that contain a change in the stability of amylase resulted from a protein mutation**.

Impact Analysis Stage consists of three classification modules. Firstly, the Disease vs. Non-Disease Classification Module separates abstracts into two groups. If the user is interested only in disease related abstracts, the abstracts that are classified as disease related are displayed on the web with a list of protein mutations. On the other hand, non-disease related abstracts are queried to Change vs. No Change Classification Module and the ones that do not contain any change in an enzyme's functionality or stability are eliminated. Lastly, in the Stability vs. Catalytic Classification Module, the remaining abstracts were sub-classified into two groups: ones that contain a change in stability of the protein structure, for those involved in catalytic activity as shown in Figure 2 and 3.

In all the classification modules of the Impact Analysis Stage, Rainbow was used as a document classifier. It assigns a document to the class with the highest score. However, the scores of the other classes are also given in the results. In the classification modules such as Disease vs. Not Disease Classification and Change vs. No Change, the abstracts in the test set are assigned to the class with the highest classification score. On the other hand, for the Stability vs. Catalytic Classification Module, before assigning an abstract to one of the classes, the difference between the scores of the two classes was checked. If the difference is less than 0.05, such abstract was assigned to both classes, because an abstract may contain a change in both the stability as well as the catalytic activity. Furthermore, it is to be noted that in the classification modules, all possible words from the vocabulary are adapted as a feature during the classification.

### Implementation

All the steps at the Preprocessing Stage are developed using Flex [38] and Bison [39]. In the Mutation Extraction Module, all regular expressions were compiled with the help of a C++ regular expression library. The Disambiguation Module makes use of Rainbow [37] in order to identify the abstracts that contain potentially ambiguous mutations. Similarly, Rainbow was used as a document classifier in all the classification modules of the Impact Analysis Stage.

During the steps shown in the Figure 1, all necessary information for the downloaded abstracts are inserted into a database. Whenever an user queries to find impacts of mutations on an enzyme's stability and functionality, information relevant to that specific enzyme can be retrieved from the system and displayed on the Web (Figure 2, 3).

### Testing

In order to measure the performance of EnzyMiner, the system was employed with the query enzymes "lipase" and "amylase". These two enzymes perform essential roles in human digestion system, and deficiency of these enzymes may lead to disorders in human body. Moreover, they have many applications in chemical and food industry. Therefore, it is easy to find both disease related and non-disease related abstracts that contain one of queried enzymes. As indicated before, possible variations of the term "mutation" was included into our search query while developing the EnzyMiner system. However, in the evaluation step, only the PubMed abstracts that contain the terms "mutation" or "mutations", and either one of the queried enzymes were downloaded. As a result, our experimental dataset showed 393 abstracts that contain the term "lipase" and 126 abstracts showed the term "amylase". All of the abstracts are manually curated by the experts for three classification schemes.

The Impact Analysis Stage of EnzyMiner consists of 3 classification modules: (a) Disease vs. Non-Disease Classification, (b) Change vs. No Change Classification, and (c) Stability vs. Catalytic Classification Modules. Before detailing the experiments performed at this stage, stating the classification algorithms and evaluation metrics used in these modules will provide better understanding of the evaluation.

#### Classification algorithms and processing options

In the classification stages of EnzyMiner, Rainbow [37] was used for document classification. It provides several classification algorithms and processing options. In order to decide which classification algorithm was more suitable, the classification performances of four algorithms (Naive Bayes, SVM, Probabilistic Indexing and Rocchio with TF-IDF weighting) were investigated. Moreover, since some of these algorithms adopt the bag-of-words approach, eight different processing models were built to observe the impact of tokenization, stemming, and the use of n-grams. In these eight models, the effect of stemming, removing commonly observed morphological and inflectional suffixes from words, and the effect of using unigrams and bigrams were tested. In addition, to observe the effect of tokenization, two types of procedures were used, these are, white space tokenizer and alphanumeric tokenizer. The white space tokenizer delimits the tokens by a whitespace only, while the alphanumeric tokenizer delimits the tokens by only nonalphanumeric characters [37].

#### Evaluation in the classification modules

As stated before, systematic evaluations for mutation extraction systems are developed with a precise definition of evaluation metrics. Although not many systems still analyze the impact of mutations, evaluation metrics for such systems were also defined. Since, the general idea for impact analysis is to perform information extraction, the standard precision, recall and F-measure are ideal parameters for evaluation [19]. We decided to use also the accuracy measure in our evaluations because we approached to this problem as a document classification problem.

Furthermore, in order to test the effectiveness of the classification modules, we followed the train-and-test and k-fold cross validation approaches together. For each classification module, 20% of the abstracts were chosen as a test set. The remaining abstracts were used as a training set and the models are trained only with these abstracts. First of all, using only the training set, 3 fold classification was performed fifty times and the average accuracy was represented as the training set accuracy. This step was repeated for all four classification algorithms and eight models. The classification algorithm with the highest accuracy model was used in the test step, where the abstracts from the test set were queried for classification. The overall accuracy of this step was represented as the test set accuracy.

## Results

### Mutation extraction stage

In order to measure the performance of Mutation Extraction Stage, all of the downloaded abstracts that contained the enzyme "lipase" or "amylase" were queried to Mutation Extraction and Disambiguation Modules of MuGeX. Out of 519 abstracts, 194 were identified to have containing the amino-acid-level mutation with 95.6% precision, 87.8% recall, 91.5 F-measure and 93.1% accuracy. These 194 abstracts were then used to evaluate the Impact Analysis Stage, where 80% of them were used as training set and the remaining as the test set. The number of abstracts in the training and test set for each classification module can be found in Tables 1, 2, 3.

**Table 1:** Experimental results of Disease vs. Non-Disease Classification Module

Disease vs. Non-Disease Classification					
	# of Abstracts	Precision	Recall	F-measure	Accuracy

Train Set	155	97.5%	93.1%	95.2	95%
Test Set	39	100%	100%	100	100%

**Table 2:** Experimental results of Change vs. No Change Classification Module

Change vs. No Change Classification					
	# of Abstracts	Precision	Recall	F-measure	Accuracy

Train Set	91	99.1%	91.9%	95.4	91.3%
Test Set	24	100%	91.3%	95.5	91.7%

**Table 3:** Experimental results of Stability vs. Catalytic Classification Module

Stability vs. Catalytic Classification					
	# of Abstracts	Precision	Recall	F-measure	Accuracy

Train Set	58	98.4%	94.9%	96.6	94.4%
Test Set	14	100%	100%	100	100%

### Impact analysis stage

**Figure 4:**
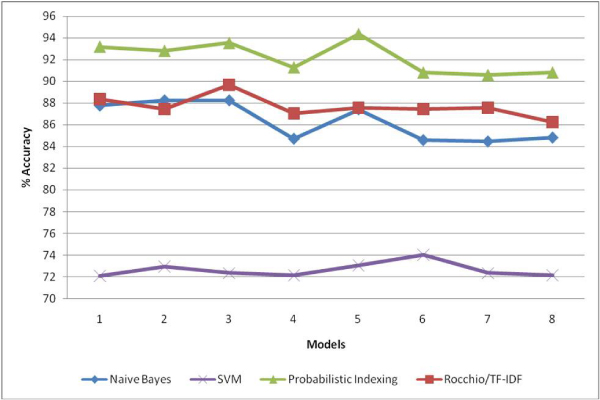
**Performances of four classification algorithms at Stability vs. Catalytic Classification Module**. Model 1: White space tokenizer, no stemming, unigram. Model 2: Alphanumeric tokenizer, no stemming, unigram. Model 3: White space tokenizer, stemming, unigram. Model 4: White space tokenizer, no stemming, bigram. Model 5: Alphanumeric tokenizer, stemming, unigram. Model 6: Alphanumeric tokenizer, no stemming, bigram. Model 7: White space tokenizer, stemming, bigram. Model 8: Alphanumeric tokenizer, stemming, bigram.

As indicated previously, performances of Naive Bayes, SVM, Probabilistic Indexing and Rocchio with TF-IDF weighting classification algorithms were investigated. The experimental runs showed that, out of four classification algorithms, Probabilistic Indexing always performed better than the other three algorithms. As shown in Figure 4, the accuracy measures of the four algorithms were compared with respect to the training set accuracies of Stability vs. Catalytic Classification Module. Probabilistic Indexing outperformed the other algorithms, while Rocchio with TF-IDF weighting performs moderately better than Naive Bayes. Although SVM is one of the best classification algorithms in document classification, its' performance is dependent on the context of the document [27]. In this application context, out of the four classification algorithms, SVM was the one with the worst performance measure. The same order was observed in the other classification modules and therefore, Probabilistic Indexing was chosen for all of the classification tasks performed in the Impact Analysis Stage.

**Figure 5:**
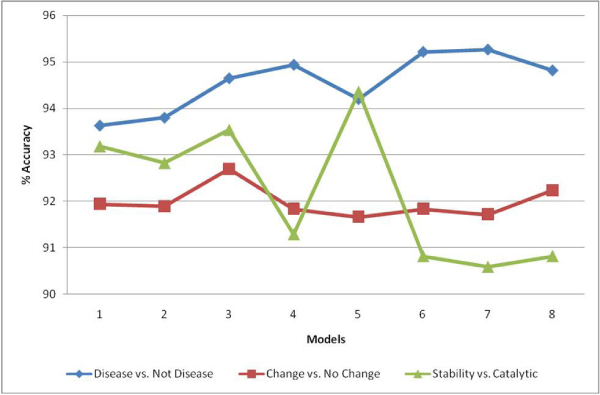
**Performance of Probabilistic Indexing with different processing options**. Model 1: White space tokenizer, no stemming, unigram. Model 2: Alphanumeric tokenizer, no stemming, unigram. Model 3: White space tokenizer, stemming, unigram. Model 4: White space tokenizer, no stemming, bigram. Model 5: Alphanumeric tokenizer, stemming, unigram. Model 6: Alphanumeric tokenizer, no stemming, bigram. Model 7: White space tokenizer, stemming, bigram. Model 8: Alphanumeric tokenizer, stemming, bigram.

As in the Disease vs. Non-Disease Classification Module, downloaded abstracts that contained at least one protein mutation were classified as disease related or non-disease related abstracts. In our experimental data, 194 abstracts that contained amino-acid-level mutation were randomly divided into training and test sets, and 3 fold classification was performed fifty times on the training set. As seen in Figure 5, the highest training set accuracy measure of 95% was obtained using Model 7 (White space tokenizer, stemming and bigram) with 95.2 F-measure. This high accuracy is expected since most of the disease related abstracts contained words such as "disease", "patient", "deficiency" or "subject", and increasing the frequency of these words by performing stemming also increases our accuracy. Moreover, word pairs such as "binding site" and "substrate specificity" are terms related to the activity of an enzyme and they were mostly observed in abstracts that are not disease related. Therefore, using bi-grams also increased our classification accuracy. After the experiments from the training set, all the abstracts in the test set were classified using Probabilistic Indexing algorithm which was trained with the above model. At the end of the classification, 100% of the abstracts were correctly classified (Table 1).

After the Disease vs. Non-Disease Classification, if the user specifically indicates an interest in a disease related abstract, the abstracts that are assigned to this class are displayed on the web with a list of protein mutations they contain. If we specifically look at the mutation extraction performance on these abstracts, our accuracy was 92.2% with 95.2% precision, 86.8% recall and 90.7 F-measure. On the other hand, if the user is not interested in a disease related abstract, the abstracts that are assigned to the non-disease related class were then continued to be processed in Change vs. No Change Classification Module.

In this module, another classification identifies the abstracts that do not contain any change in the enzyme's stability or functionality. As shown in Figure 5, the highest classification accuracy of the training set was obtained using Model 3 (White space tokenizer, stemming, unigram) with 91.3% accuracy and 95.4 F-measure, and the results of this experiment are given in Table 2. Using this model, 91.7% accuracy and 95.5 F-measure were obtained in the classification of the test set and at the end of this step, abstracts that were classified as no change are eliminated.

In the last step, the remaining abstracts are classified into two groups, these are, abstracts that contain information on a change in the enzyme's stability, or abstracts that discuss the changes in the enzyme's catalytic activity. When this classification was performed on the training set, the highest accuracy measure 94.4% and 96.6 F-measure were observed with Model 5 (Alphanumeric tokenizer, stemming, unigram), and thus a classification was performed on the test set using this model. At the end, it was observed that all the abstracts in the test set were correctly classified. As illustrated in Table 3, the experimental results of both the training and test sets are shown to be very high, which is expected since the classification model successfully identifies the most informative words such as "thermostability", "pH", "temperature", "sensitivity" and "specificity" as well as increase their frequency with stemming.

It has been observed that classification modules respond to the different processing options in a more or less similar way. However, highest training set accuracies were observed in different models for each classification module. It shows that processing options have different effects on the classification of different concept. The only common part of these three models was stemming, which is an expected result, since it reduces the dimensionality of the term space which is a good thing for systems that have small training sets [27] such as EnzyMiner.

As indicated before, our experimental runs were performed on the abstracts that are obtained using the words "lipase" and "amylase". All three classification modules were first trained with abstracts that contain "lipase", and tested with abstracts that contain "amylase" later. This was performed in order to prove that the success of EnzyMiner was not dependent on the inclusion of the query enzyme abstracts in the training set. In our test set we had 45 abstracts that contain a protein mutation and the term "amylase". Out of these 45 abstracts, 43 of them were non-disease related, out of these 43 non-disease related abstracts, 40 of them contained a change in the enzyme's stability or functionality. The results of these experiments as shown in Table 4 indicate that training EnzyMiner on lipases provided necessary information to correctly classify the amylases. This proves that training our system with only one enzyme is sufficient to obtain high classification accuracies for the other enzymes, which is the intended use of EnzyMiner.

**Table 4:** Experimental results on Amylases

	# of Abstracts	# of Correctly Identified Abstracts	Accuracy
Mutation Extraction	45	42	93.3%
Disease vs. Non-Disease Classification	45	41	91.1%
Change vs. No Change Classification	43	37	86%
Stability vs. Catalytic Classification	40	34	85%

## Conclusion

In this paper, we described EnzyMiner, an automated system designed to identify the PubMed abstracts that contain information on the impact of a protein level mutation on the stability and the activity of a given enzyme. To our knowledge, besides the manually curated enzyme database BRENDA, there is no tool or database that provides the same information like the EnzyMiner. Although, we performed our experiments on lipases and amylases, the EnzyMiner can be applied to other enzymes without any modification. For mutation extraction, we used MuGeX which handles ambiguous mutation citations successfully, and thus has a high accuracy and F-measure. In the case of impact analysis of mutations, EnzyMiner uses document classification first to separate disease related abstracts, then to eliminate abstracts that do not contain any change in enzyme's stability or catalytic activity, and lastly to classify the remaining abstracts according to the impact of the mutation on the stability or on the catalytic activity.

Currently, EnzyMiner does not give any information about the direction of the impact, whether there is an increase or decrease in the stability conditions or catalytic activity. Such a specification cannot be handled as a classification problem because there are abstracts that contain several mutations, each effect the stability condition or catalytic activity of an enzyme in different directions. Moreover, EnzyMiner does not specify the kind of change in the stability conditions. It cannot distinguish between the changes in pH and temperature values. Similarly, in case of functionality, it cannot differentiate a change in the enzyme's specificity from a change in the sensitivity. Another limitation of EnzyMiner is, if an abstract contains several enzymes and protein mutations, it cannot associate the enzymes to the corresponding mutations, since EnzyMiner do not use proximity measures. Therefore, in the future, natural language processing techniques should be employed in order to overcome the above limitations and extract more specific information about the impact of a mutation on an enzyme.

## Availability

The EnzyMiner system and the gold standard corpus for the Impact Analysis Stage are available under the URL .

## Competing interests

The authors declare that they have no competing interests.

## Authors' contributions

SY designed the experiments, carried out the analysis, and wrote the manuscript. US was the principal investigator of this system. He conceptualized the system architecture and helped draft the manuscript.
